# 6,6′-Dimethyl-2,2′-bipyridin-1-ium tetra­chloridoaurate(III)

**DOI:** 10.1107/S160053681202853X

**Published:** 2012-06-30

**Authors:** Marzieh Zare Dehnavi, Anita Abedi

**Affiliations:** a Department of Chemistry, North Tehran Branch, Islamic Azad University, Tehran, Iran

## Abstract

In the anion of the title compound, (C_12_H_13_N_2_)[AuCl_4_], the Au^III^ atom has a square-planar coordination. In the crystal, inter­molecular N—H⋯Cl and C—H⋯Cl hydrogen bonds and π–π contacts between the pyridine rings [centroid–centroid distance = 3.5419 (19) Å] result in the formation of a supra­molecular structure.

## Related literature

For related structures, see: Abedi *et al.* (2008 ▶, 2011 ▶); Amani *et al.* (2010 ▶); Calleja *et al.* (2001 ▶); Faza­eli *et al.* (2010 ▶); Hasan *et al.* (1999 ▶); Hojjat Kashani *et al.* (2008 ▶); Johnson & Steed (1998 ▶); Kalateh *et al.* (2008 ▶); Safari *et al.* (2009 ▶); Yap *et al.* (1995 ▶); Yıldırım *et al.* (2009*a* ▶,*b* ▶); Zhang *et al.* (2006 ▶).
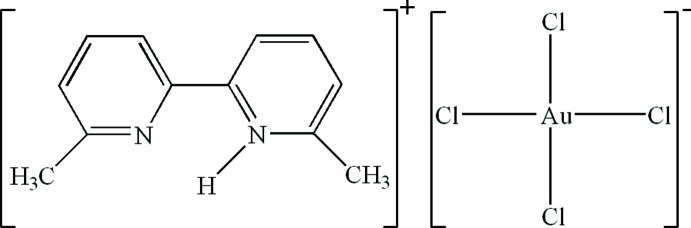


## Experimental

### 

#### Crystal data

(C_12_H_13_N_2_)[AuCl_4_]
*M* *_r_* = 524.01
Monoclinic, 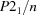

*a* = 10.8942 (9) Å
*b* = 11.6784 (10) Å
*c* = 12.2866 (11) Å
β = 95.772 (5)°
*V* = 1555.3 (2) Å^3^
*Z* = 4
Mo *K*α radiation
μ = 10.13 mm^−1^
*T* = 100 K
0.20 × 0.15 × 0.15 mm

#### Data collection

Bruker APEXII CCD diffractometer
Absorption correction: multi-scan (*SADABS*; Bruker, 2001 ▶) *T*_min_ = 0.182, *T*_max_ = 0.227
12293 measured reflections
4101 independent reflections
3699 reflections with *I* > 2σ(*I*)
*R*_int_ = 0.032

#### Refinement

*R*[*F* ^2^ > 2σ(*F* ^2^)] = 0.023
*wR*(*F* ^2^) = 0.049
*S* = 1.00
4101 reflections
174 parameters
H-atom parameters constrained
Δρ_max_ = 0.83 e Å^−3^
Δρ_min_ = −1.15 e Å^−3^

### 

Data collection: *APEX2* (Bruker, 2007 ▶); cell refinement: *SAINT* (Bruker, 2007 ▶); data reduction: *SAINT*; program(s) used to solve structure: *SHELXTL* (Sheldrick, 2008 ▶); program(s) used to refine structure: *SHELXTL*; molecular graphics: *XP* in *SHELXTL* (Sheldrick, 2008 ▶) and *Mercury* (Macrae *et al.*, 2006 ▶); software used to prepare material for publication: *SHELXTL*.

## Supplementary Material

Crystal structure: contains datablock(s) I, global. DOI: 10.1107/S160053681202853X/hy2560sup1.cif

Structure factors: contains datablock(s) I. DOI: 10.1107/S160053681202853X/hy2560Isup2.hkl

Additional supplementary materials:  crystallographic information; 3D view; checkCIF report

## Figures and Tables

**Table 1:** Hydrogen-bond geometry (Å, °)

*D*—H⋯*A*	*D*—H	H⋯*A*	*D*⋯*A*	*D*—H⋯*A*
N2—H2*N*⋯Cl3^i^	0.82	2.80	3.419 (2)	134
C4—H4*A*⋯Cl2^ii^	0.95	2.79	3.434 (4)	126

Symmetry codes: (i) 
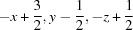
; (ii) 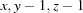.

**Fig. 1.**
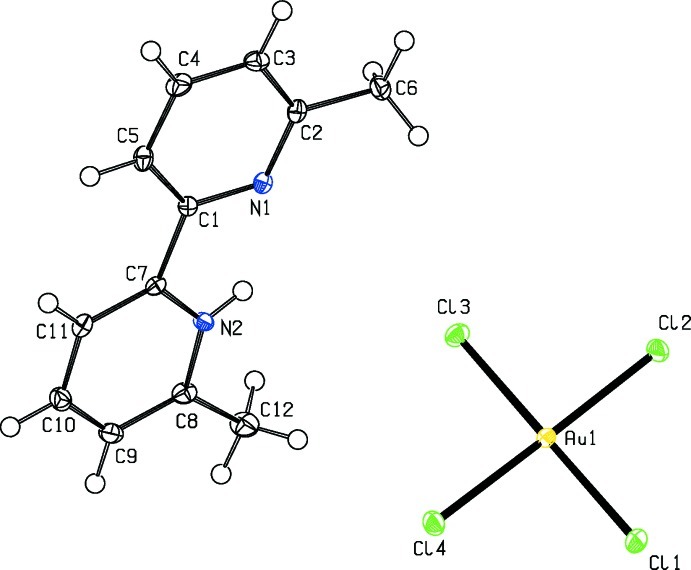
The molecular structure of the title compound. Displacement ellipsoids are drawn at the 50% probability level.

**Fig. 2.**
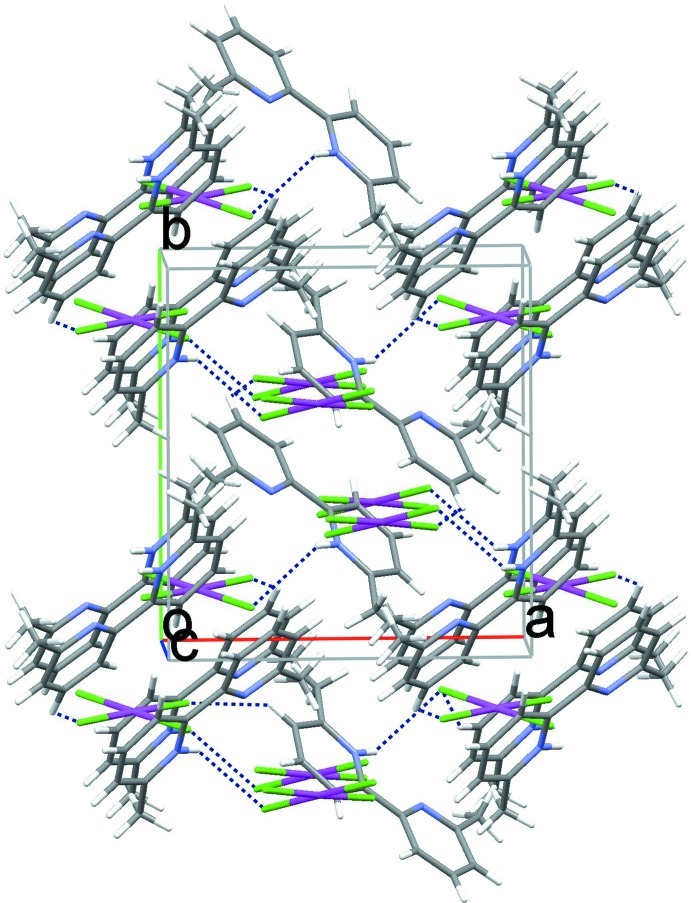
Crystal packing diagram for the title compound. Hydrogen bonds are shown as dashed lines.

## supplementary crystallographic information

### Comment

In the recent years, we reported the synthesis and crystal structures of two proton transfer complexes (Abedi *et al.*, 2008; Kalateh *et al.*, 2008). Several proton transfer systems using HAuCl_4_ as proton acceptor molecule, such as [EMI][AuCl_4_], (II), [BMI]_2_[AuCl_4_].2H_2_O, (III), (Hasan *et al.*, 1999), [H_2_bipy][AuCl_4_]Cl, (IV), (Zhang *et al.*, 2006), [H_7_O_3_][15-crown-5][AuCl_4_], (V), [H_5_O_2_][benzo-15-crown-5]_2_[AuCl_4_], (VI), (Johnson & Steed, 1998), [H_5_O_2_]_2_[12-crown-4]_2_[AuCl_4_]_2_, (VII), [H_3_O][18-crown-6][AuCl_4_], (VIII), [H_3_O][4-nitrobenzo-18-crown-6][AuCl_4_], (IX), (Calleja *et al.*, 2001), [Ph_2_py.H][AuCl_4_], (X), (Yap *et al.*, 1995), [H_2_DA18C6][AuCl_4_], (XI), (Hojjat Kashani *et al.*, 2008), [Me_2_Ph_2_phen.H][AuCl_4_], (XII), (Yıldırım *et al.*, 2009*a*), [pz(py)_2_.H][AuCl_4_], (XIII), (Yıldırım *et al.*, 2009*b*), [Phpy.H][AuCl_4_], (XIV), (Amani *et al.*, 2010), [Dafonium][AuCl_4_].[dafone], (XV), (Safari *et al.*, 2009), [tppzH_2_][AuCl_4_]_2_, (XVI), (Abedi *et al.*, 2011) and [TBA]_2_[AuCl_4_][Cl], (XVII), (Fazaeli *et al.*, 2010) (EMI = 1-ethyl-3-methylimidazolium, BMI = 1-butyl-3-methylimidazolium, H_2_bipy = 2,2'-bipyridinium, Ph_2_py.H = 2,6-diphenylpyridinium, H_2_DA18C6 = 1,10-diazonia-18-crown-6, Me_2_Ph_2_phen.H = 2,9-dimethyl-4,7-diphenyl-1,10-phenanthrolin-1-ium, pz(py)_2_.H = 2-[3-(2-pyridyl)pyrazin-2-yl]pyridinium, Phpy.H = 3-phenylpyridinium, Dafonium = 9-oxo-4,5-diazafluoren-4-ium, dafone = 4,5-diazafluoren-9-one, tppzH_2_ = 2,5-bis(pyridinium-2-yl)-3,6-bis(2-pyridyl)pyrazine and TBA = tribenzylammonium) have been synthesized and characterized by single-crystal X-ray diffraction methods. We report herein the synthesis and crystal structure of the title compound, (I).

In the anion of the title compound (Fig. 1), the Au^III^ atom has a square-planar coordination. The Au—Cl bond lengths and angles are within normal range (XII–XVII). In the crystal, intermolecular N—H···Cl and C—H···Cl hydrogen bonds (Table 1) and π–π contacts between the pyridine rings, *Cg*1···*Cg*2^i^ [*Cg*1 and *Cg*2 are the centroids of the rings N1/C1–C5 and N2/C7–C11, respectively. Symmetry code: (i) 2-*x*, -y, -*z*], with a centroid–centroid distance of 3.5419 (19) Å, are effective in the stabilization of the crystal structure, resulting in the formation of a supramolecular structure.

### Experimental

For the preparation of the title compound, a solution of 6,6'-dimethyl-2,2'-bipyridine (0.21 g, 1.11 mmol) in methanol (10 ml) was added to a solution of HAuCl_4_.3H_2_O, (0.58 g, 1.11 mmol) in acetonitrile (10 ml) and the resulting yellow solution was stirred for 15 min at 313 K. This solution was left to evaporate slowly at room temperature. After one week, yellow prismatic crystals of the title compound were isolated (yield: 0.46 g, 79.1%; m. p. 453 K).

### Refinement

H atom of NH group was found in a difference Fourier map and refined as riding, with *U*_iso_(H) = 1.2*U*_eq_(N). H atoms on C atoms were positioned geometrically and refined as riding atoms, with C—H = 0.95 (aromatic) and 0.98 (methyl) Å and with *U*_iso_(H) = 1.2(1.5 for methyl)*U*_eq_(C).

### Crystal data

**Table:**

(C_12_H_13_N_2_)[AuCl_4_]	*F*(000) = 984
*M**_r_* = 524.01	*D*_x_ = 2.238 Mg m^−^^3^
Monoclinic, *P*2_1_/*n*	Mo *K*α radiation, λ = 0.71073 Å
Hall symbol: -P 2yn	Cell parameters from 1542 reflections
*a* = 10.8942 (9) Å	θ = 3.0–30.0°
*b* = 11.6784 (10) Å	µ = 10.13 mm^−^^1^
*c* = 12.2866 (11) Å	*T* = 100 K
β = 95.772 (5)°	Prism, yellow
*V* = 1555.3 (2) Å^3^	0.20 × 0.15 × 0.15 mm
*Z* = 4	

### Data collection

**Table:**

Bruker APEXII CCD diffractometer	4101 independent reflections
Radiation source: fine-focus sealed tube	3699 reflections with *I* > 2σ(*I*)
Graphite monochromator	*R*_int_ = 0.032
φ and ω scans	θ_max_ = 29.0°, θ_min_ = 2.4°
Absorption correction: multi-scan (*SADABS*; Bruker, 2001)	*h* = −14→14
*T*_min_ = 0.182, *T*_max_ = 0.227	*k* = −15→15
12293 measured reflections	*l* = −16→16

### Refinement

**Table:**

Refinement on *F*^2^	Primary atom site location: structure-invariant direct methods
Least-squares matrix: full	Secondary atom site location: difference Fourier map
*R*[*F*^2^ > 2σ(*F*^2^)] = 0.023	Hydrogen site location: mixed
*wR*(*F*^2^) = 0.049	H-atom parameters constrained
*S* = 1.00	*w* = 1/[σ^2^(*F*_o_^2^) + (0.0228*P*)^2^ + 0.3281*P*] where *P* = (*F*_o_^2^ + 2*F*_c_^2^)/3
4101 reflections	(Δ/σ)_max_ = 0.002
174 parameters	Δρ_max_ = 0.83 e Å^−^^3^
0 restraints	Δρ_min_ = −1.15 e Å^−^^3^

### Special details

**Table:**

Geometry. All e.s.d.'s (except the e.s.d. in the dihedral angle between two l.s. planes) are estimated using the full covariance matrix. The cell e.s.d.'s are taken into account individually in the estimation of e.s.d.'s in distances, angles and torsion angles; correlations between e.s.d.'s in cell parameters are only used when they are defined by crystal symmetry. An approximate (isotropic) treatment of cell e.s.d.'s is used for estimating e.s.d.'s involving l.s. planes.
Refinement. Refinement of *F*^2^ against ALL reflections. The weighted *R*-factor *wR* and goodness of fit *S* are based on *F*^2^, conventional *R*-factors *R* are based on *F*, with *F* set to zero for negative *F*^2^. The threshold expression of *F*^2^ > σ(*F*^2^) is used only for calculating *R*-factors(gt) *etc*. and is not relevant to the choice of reflections for refinement. *R*-factors based on *F*^2^ are statistically about twice as large as those based on *F*, and *R*- factors based on ALL data will be even larger.

### Fractional atomic coordinates and isotropic or equivalent isotropic displacement parameters (Å^2^)

**Table:**

	*x*	*y*	*z*	*U*_iso_*/*U*_eq_	
Au1	0.910926 (11)	0.846230 (10)	0.536181 (9)	0.00895 (4)	
Cl1	1.05716 (8)	0.80777 (8)	0.67847 (7)	0.01993 (18)	
Cl2	0.76133 (7)	0.82538 (7)	0.65184 (6)	0.01523 (16)	
Cl3	0.76425 (7)	0.88766 (7)	0.39541 (6)	0.01501 (16)	
Cl4	1.05950 (7)	0.86081 (7)	0.41815 (7)	0.01552 (16)	
N1	0.7935 (2)	0.0942 (2)	0.0534 (2)	0.0096 (5)	
N2	0.9773 (2)	0.2313 (2)	0.0264 (2)	0.0102 (5)	
H2N	0.9224	0.2323	0.0676	0.012*	
C1	0.8500 (3)	0.0735 (3)	−0.0370 (2)	0.0089 (6)	
C2	0.6953 (3)	0.0310 (3)	0.0722 (2)	0.0107 (6)	
C3	0.6523 (3)	−0.0561 (3)	0.0007 (3)	0.0136 (6)	
H3A	0.5832	−0.1010	0.0158	0.016*	
C4	0.7107 (3)	−0.0771 (3)	−0.0928 (3)	0.0154 (7)	
H4A	0.6818	−0.1361	−0.1422	0.018*	
C5	0.8112 (3)	−0.0112 (3)	−0.1130 (3)	0.0131 (6)	
H5A	0.8526	−0.0231	−0.1766	0.016*	
C6	0.6349 (3)	0.0588 (3)	0.1736 (3)	0.0146 (7)	
H6A	0.6978	0.0635	0.2363	0.022*	
H6B	0.5754	−0.0013	0.1867	0.022*	
H6C	0.5920	0.1325	0.1640	0.022*	
C7	0.9564 (3)	0.1483 (3)	−0.0502 (3)	0.0098 (6)	
C8	1.0677 (3)	0.3099 (3)	0.0276 (3)	0.0129 (6)	
C9	1.1446 (3)	0.3059 (3)	−0.0547 (3)	0.0144 (7)	
H9A	1.2088	0.3605	−0.0571	0.017*	
C10	1.1274 (3)	0.2212 (3)	−0.1343 (3)	0.0148 (7)	
H10A	1.1808	0.2183	−0.1908	0.018*	
C11	1.0340 (3)	0.1407 (3)	−0.1328 (3)	0.0124 (6)	
H11A	1.0234	0.0822	−0.1867	0.015*	
C12	1.0788 (3)	0.3951 (3)	0.1189 (3)	0.0185 (7)	
H12A	1.1431	0.4510	0.1067	0.028*	
H12B	1.1007	0.3555	0.1885	0.028*	
H12C	0.9999	0.4348	0.1215	0.028*	

### Atomic displacement parameters (Å^2^)

**Table:**

	*U*^11^	*U*^22^	*U*^33^	*U*^12^	*U*^13^	*U*^23^
Au1	0.00970 (6)	0.00822 (7)	0.00884 (6)	0.00079 (4)	0.00044 (4)	−0.00109 (4)
Cl1	0.0138 (4)	0.0335 (5)	0.0120 (4)	0.0015 (4)	−0.0011 (3)	0.0048 (3)
Cl2	0.0142 (4)	0.0191 (4)	0.0129 (3)	−0.0038 (3)	0.0041 (3)	−0.0045 (3)
Cl3	0.0141 (4)	0.0159 (4)	0.0143 (3)	0.0012 (3)	−0.0021 (3)	0.0022 (3)
Cl4	0.0134 (4)	0.0201 (4)	0.0136 (4)	0.0027 (3)	0.0039 (3)	0.0017 (3)
N1	0.0119 (13)	0.0076 (13)	0.0093 (12)	0.0009 (10)	0.0004 (10)	0.0012 (10)
N2	0.0088 (12)	0.0109 (13)	0.0113 (12)	−0.0010 (10)	0.0022 (10)	−0.0012 (10)
C1	0.0084 (14)	0.0084 (14)	0.0098 (13)	0.0040 (11)	0.0010 (11)	0.0008 (11)
C2	0.0134 (15)	0.0090 (15)	0.0096 (13)	0.0033 (12)	0.0002 (11)	0.0027 (12)
C3	0.0116 (15)	0.0122 (16)	0.0167 (15)	−0.0024 (13)	−0.0002 (12)	0.0004 (13)
C4	0.0167 (16)	0.0137 (16)	0.0153 (15)	−0.0034 (13)	−0.0009 (13)	−0.0062 (13)
C5	0.0175 (16)	0.0119 (16)	0.0099 (14)	0.0000 (13)	0.0022 (12)	−0.0024 (12)
C6	0.0161 (16)	0.0156 (17)	0.0125 (15)	−0.0028 (13)	0.0036 (12)	0.0015 (13)
C7	0.0099 (14)	0.0092 (15)	0.0099 (14)	0.0004 (12)	−0.0013 (11)	0.0001 (12)
C8	0.0138 (15)	0.0088 (15)	0.0157 (15)	−0.0023 (13)	−0.0009 (12)	0.0009 (12)
C9	0.0104 (15)	0.0160 (17)	0.0167 (16)	−0.0015 (13)	0.0004 (13)	0.0040 (13)
C10	0.0125 (15)	0.0185 (17)	0.0137 (15)	0.0027 (13)	0.0027 (12)	0.0033 (13)
C11	0.0143 (15)	0.0129 (16)	0.0098 (14)	0.0010 (13)	−0.0006 (12)	−0.0008 (12)
C12	0.0186 (17)	0.0157 (17)	0.0215 (17)	−0.0050 (14)	0.0032 (14)	−0.0072 (14)

### Geometric parameters (Å, º)

**Table:**

Au1—Cl2	2.2804 (8)	C4—H4A	0.9500
Au1—Cl3	2.2854 (8)	C5—H5A	0.9500
Au1—Cl4	2.2856 (8)	C6—H6A	0.9800
Au1—Cl1	2.2882 (8)	C6—H6B	0.9800
N1—C2	1.339 (4)	C6—H6C	0.9800
N1—C1	1.345 (4)	C7—C11	1.388 (5)
N2—C8	1.345 (4)	C8—C9	1.378 (5)
N2—C7	1.353 (4)	C8—C12	1.496 (5)
N2—H2N	0.8221	C9—C10	1.390 (5)
C1—C5	1.396 (4)	C9—H9A	0.9500
C1—C7	1.474 (4)	C10—C11	1.387 (5)
C2—C3	1.395 (4)	C10—H10A	0.9500
C2—C6	1.502 (4)	C11—H11A	0.9500
C3—C4	1.390 (5)	C12—H12A	0.9800
C3—H3A	0.9500	C12—H12B	0.9800
C4—C5	1.382 (5)	C12—H12C	0.9800
			
Cl2—Au1—Cl3	90.29 (3)	C2—C6—H6B	109.5
Cl2—Au1—Cl4	178.02 (3)	H6A—C6—H6B	109.5
Cl3—Au1—Cl4	89.42 (3)	C2—C6—H6C	109.5
Cl2—Au1—Cl1	89.38 (3)	H6A—C6—H6C	109.5
Cl3—Au1—Cl1	179.00 (3)	H6B—C6—H6C	109.5
Cl4—Au1—Cl1	90.94 (3)	N2—C7—C11	118.8 (3)
C2—N1—C1	118.9 (3)	N2—C7—C1	115.3 (3)
C8—N2—C7	124.6 (3)	C11—C7—C1	125.8 (3)
C8—N2—H2N	124.1	N2—C8—C9	117.8 (3)
C7—N2—H2N	110.9	N2—C8—C12	117.8 (3)
N1—C1—C5	123.1 (3)	C9—C8—C12	124.3 (3)
N1—C1—C7	114.5 (3)	C8—C9—C10	119.5 (3)
C5—C1—C7	122.4 (3)	C8—C9—H9A	120.3
N1—C2—C3	121.1 (3)	C10—C9—H9A	120.3
N1—C2—C6	116.6 (3)	C11—C10—C9	121.3 (3)
C3—C2—C6	122.2 (3)	C11—C10—H10A	119.3
C4—C3—C2	119.8 (3)	C9—C10—H10A	119.3
C4—C3—H3A	120.1	C10—C11—C7	117.9 (3)
C2—C3—H3A	120.1	C10—C11—H11A	121.1
C5—C4—C3	119.2 (3)	C7—C11—H11A	121.1
C5—C4—H4A	120.4	C8—C12—H12A	109.5
C3—C4—H4A	120.4	C8—C12—H12B	109.5
C4—C5—C1	117.8 (3)	H12A—C12—H12B	109.5
C4—C5—H5A	121.1	C8—C12—H12C	109.5
C1—C5—H5A	121.1	H12A—C12—H12C	109.5
C2—C6—H6A	109.5	H12B—C12—H12C	109.5
			
C2—N1—C1—C5	0.0 (4)	N1—C1—C7—N2	−3.9 (4)
C2—N1—C1—C7	179.6 (3)	C5—C1—C7—N2	175.6 (3)
C1—N1—C2—C3	0.8 (4)	N1—C1—C7—C11	176.4 (3)
C1—N1—C2—C6	−179.2 (3)	C5—C1—C7—C11	−4.1 (5)
N1—C2—C3—C4	−1.0 (5)	C7—N2—C8—C9	0.0 (5)
C6—C2—C3—C4	179.1 (3)	C7—N2—C8—C12	−179.4 (3)
C2—C3—C4—C5	0.3 (5)	N2—C8—C9—C10	−0.8 (5)
C3—C4—C5—C1	0.6 (5)	C12—C8—C9—C10	178.6 (3)
N1—C1—C5—C4	−0.7 (5)	C8—C9—C10—C11	0.3 (5)
C7—C1—C5—C4	179.7 (3)	C9—C10—C11—C7	1.0 (5)
C8—N2—C7—C11	1.3 (5)	N2—C7—C11—C10	−1.8 (4)
C8—N2—C7—C1	−178.4 (3)	C1—C7—C11—C10	177.9 (3)

### Hydrogen-bond geometry (Å, º)

**Table:**

*D*—H···*A*	*D*—H	H···*A*	*D*···*A*	*D*—H···*A*
N2—H2*N*···Cl3^i^	0.82	2.80	3.419 (2)	134
C4—H4*A*···Cl2^ii^	0.95	2.79	3.434 (4)	126

Symmetry codes: (i) −*x*+3/2, *y*−1/2, −*z*+1/2; (ii) *x*, *y*−1, *z*−1.
